# Drug Hypersensitivity and Anaphylaxis in Cancer and Chronic Inflammatory Diseases: The Role of Desensitizations

**DOI:** 10.3389/fimmu.2017.01472

**Published:** 2017-11-08

**Authors:** Mariana Castells

**Affiliations:** ^1^Allergy and Immunology, Brigham and Women’s Hospital, Harvard Medical School, Boston, MA, United States

**Keywords:** desensitization, monoclonal antibodies in cancer, platins, drug allergy, taxanes

## Abstract

Drug allergy is a rising problem in the twenty-first century which affects all populations and races, children, and adults, and for which the recognition, diagnosis, management, and treatment is still not well standardized. Classical and new chemotherapy drugs, monoclonal antibodies (MoAbs), and small molecules to treat cancer and chronic inflammatory diseases are aimed at improving quality of life and life expectancy of patients, but an increasing number of reactions including anaphylaxis precludes their use in targeted populations. Women are more affected by drug allergy and up to 27% of women with ovarian and breast cancer develop carboplatin allergy after multiple cycles of treatment. Carriers of BRCA genes develop drug allergy after fewer exposures and can present with severe reactions, including anaphylaxis. Atopic patients are at increased risk for chemotherapy and MoAbs drug allergy and the current patterns of treatment with recurrent and intermittent drug exposures may favor the development of drug allergies. To overcome drug allergy, desensitization has been developed, a novel approach which provides a unique opportunity to protect against anaphylaxis and to improve clinical outcomes. There is evidence that inhibitory mechanisms blocking IgE/antigen mast cell activation are active during desensitization, enhancing safety. Whether desensitization modulates drug allergic and anaphylactic responses facilitating tolerance is currently being investigated. This review provides insight into the current knowledge of drug allergy and anaphylaxis to cancer and chronic inflammatory diseases drugs, the mechanisms of drug desensitization and its applications to personalized medicine.

## Introduction

With the unprecedented use of chemotherapies drugs and targeted monoclonal antibodies (MoAbs) and small molecules in the twenty-first century, increased hypersensitivity reactions (HSRs) have emerged worldwide (1, 2). Drug allergic reactions are unexpected, can be severe including anaphylaxis and prevent the use of first-line therapies, with consequent impact in patient’s survival and quality of life (3, 4). These reactions range from mild cutaneous manifestations such as pruritus and hives to life-threatening anaphylaxis with hypotension, oxygen desaturation and cardiovascular collapse, and deaths have been reported after re-exposure to allergic drugs (5, 6). The presentation of symptoms can be atypical such as pain, which has been associated with taxenes reactions, and chills and fever which have been seen with oxaliplatin and MoAbs reactions (7, 8). Delayed reactions occurring more than 24 h after chemotherapy infusions can be due to the prolonged half-life of MoAbs and the presence of premedications, which may mask the acute phase of the reactions (9, 10).

The traditional classification of drug hypersensitivity and allergy into the classical types I–IV does not encompass the current spectrum of reactions and symptoms occurring in cancer patients and patients with chronic inflammatory diseases (11, 12). Some of the reactions have no known underlying mechanism, others have a known mechanism which is not part of the four described types and some drugs can induce mixed reactions with two or more proposed mechanisms (13, 14). Hypersensitivity to rituximab, a chimeric anti-CD20 MoAb, can induce cytokine-like reactions with chills, fevers, hypotension, and oxygen desaturation, which have been attributed to the release of cytokines such as IL-6, IL-1β, and TNF-α and are named cytokine release syndrome or cytokine storm, which is not contemplated in the Gell and Coombs classification (15). In contrast, some patients have classical IgE-mediated reactions to rituximab and have presented positive skin testing demonstrating that IgE and mast cells are part of the underlying mechanism (16). Some patients reactive to oxaliplatin present mixed reactions with Type I features such as hives and hypotension, along with fever and chills as seen in cytokine storm-like reactions, presenting a complex mixed pattern of reactivity which provides challenges to management and treatment (17). During mixed reactions tryptase, the major mast cell protease, and IL-6 can be elevated in serum indicating mast cell activation and cytokine release from unknown cellular sources. Reactions to taxenes can trigger direct mast cell/basophil activation with elevation of serum tryptase with or without evidence of IgE, indicating that more than one mechanism can explain taxane hypersensitivity. A different receptor than FceRI, such as the recently described MrgprX2 for drugs with THIQ motifs such as quinolones and paralyzing agents such as atracuronium could be activated during non-IgE taxane reactions (13, 18–20).

Patients presenting with delayed cutaneous reactions are at a great concern for Stevens–Johnson syndrome and toxic epidermal necrolysis, two life-threatening conditions which can lead to permanent disability, blindness, and dramatic decrease in the quality of life for survivors (16, 21). The underlying mechanisms of the reactions are poorly understood and up to now no predictive markers have been available. Genetic susceptibility and defined HLA haplotypes are thought to be risk factors for some of the reactions, such as HLA-B 5701 in HIV patients reactive to abacavir. In patients with targeted haplotypes, a new role for viral reactivation of HHV6 and other virus have been demonstrated, and the pathogenic role of the virus is under study (22–24).

To provide an operational classification which can adapt to the increasing knowledge of the mechanisms of reactions and to the symptoms and clinical presentations, a recent initiative has provided a new terminology, applicable to precision medicine. In the new categorization drug allergy phenotypes are defined by the underlying endotypes and associated biomarkers and can be used in personalized medicine, with each patient being categorized according to her/his symptoms complex presentation. Current phenotypes include acute and delayed reactions with IgE and non-IgE involvement, cytokine storm, and mixed patterns. The endotypes responsible for the expression of symptoms include mast cell and basophil activation through known receptors (FceRI, FcgR, MRGPRX2) and directly through known receptors: complement, kinin and bradykinin activation and COX-1 inhibition. Associated biomarkers include serum tryptase, skin testing, basophil activation test, specific IgE and patch testing among others (13, 25–28).

Patients presenting with reactions compatible with phenotypes consistent with acute and delayed IgE and non-IgE, mast cell/basophil activation, and T cell activation endoptypes may be prevented from the use of first-line therapies for fear of inducing anaphylaxis or more severe delayed reactions upon re-exposure to the allergenic drug. A groundbreaking procedure, desensitization, has emerged in the last 15 years as a proven effective and safe procedure to maintain patients on their first-line medications.

## Clinical Vignette

Mrs. MFF is a 49-year-old healthy female who was discovered to have ovarian cancer after a routine gynecology ultrasound and was initially treated with surgery and chemotherapy with six courses of carboplatin and paclitaxel and entered remission. Two years later, the CA125 is increased and new masses are found in her abdomen, a diagnosis of recurrent stage 4 ovarian cancer is made and carboplatin and paclitaxel restarted. After the second course of carboplatin, the patient feels her hands itchy but finished the infusion and did not have any further symptoms. On the day of her third infusion, the patient presented flushing, generalized pruritus, shortness of breath, and sudden dizziness. The blood pressure drops below normal range as well as the oxygen saturation and the patient has a syncopal episode and needs to be resuscitated with epinephrine, fluids, anti-histamines, and steroids. She recovers and her diagnosis is of anaphylaxis, a serum tryptase level during the episode is elevated at 52 ng/ml (normal range 11.4 ng/ml). The patient is evaluated for carboplatin allergy and skin testing is positive. Her options are to change to a second-line agent which is likely to reduce her life expectancy or to remain on first-line therapy with carboplatin but because of her anaphylactic reaction this option is not considered safe unless carboplatin can be introduced through desensitization, a powerful and novel intervention which has shown to protect patients against anaphylaxis and permit re-introduction of allergy drugs.

## Drug Desensitization

The term drug desensitization is currently used to define a process by which a patient’s immune response to a drug is modified to generate temporary tolerance, taking advantage of well characterized inhibitory pathways (6). In the case of IgE-mediated drug allergy, positive skin testing and specific serum IgE can be used as biomarkers along with elevated serum tryptase level during the acute reaction (8, 11, 17). Patients without evidence of IgE mechanism are good candidates for desensitization provided the phenotype of the drug reaction is a type I or a type IV like reaction without features of SJS/TEN (16, 21, 29) (Table 1).

**Table 1:** Indications, contraindications and risk factors for drug desensitization.

Indications	High-risk patients	Contraindications
Reactions type I (mast cells/IgE/basofills) Reaction type IV (except SCARs)	Severe anaphylaxis (intubation)	Severe cutaneous adverse reactions (SCARs) (SJS/TEN, DIHS/DRESS, AGEP)
No alternative drug	Severe respiratory disease	Immunocytotoxic reactions (type II reactions)
Drug is more effective and/or associated with less side effects	Severe cardiac disease	Vasculitis
Drug has a unique mechanism of action	Severe systemic diseases	Serum sickness-like (type III reactions)
	Use of beta-blockers, ACE inhibitors	
	Pregnancy	

*SJS, Stevens–Johnson syndrome; TEN, toxic epidermal necrolysis; DIHS, drug-induced hypersensitivity syndrome; DRESS, drug reaction (rash) with eosinophilia and systemic symptoms; AGEP, acute generalized exanthematous*.

**Figure 1:**
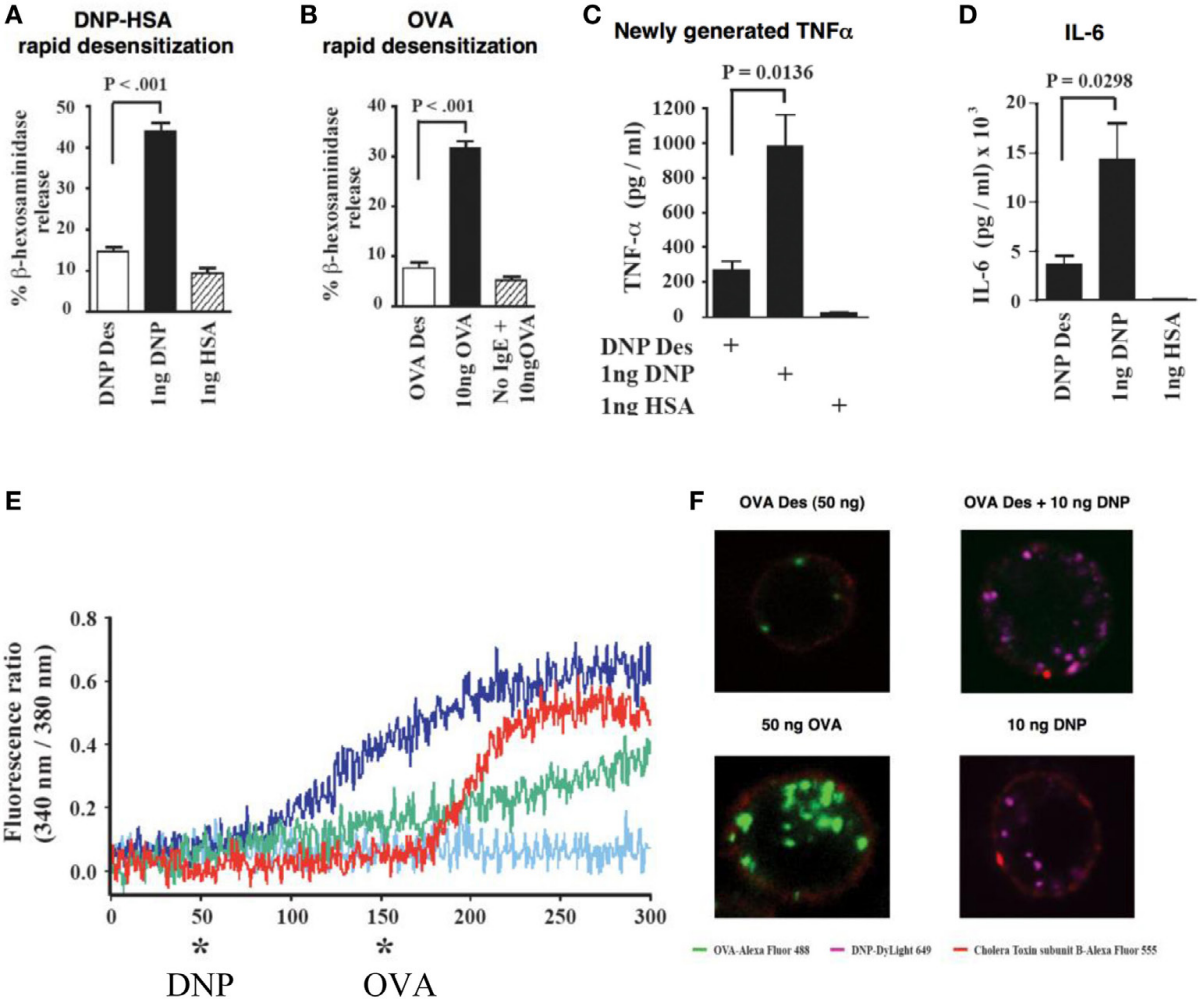
*In vitro* IgE/antigen mouse mast cells activation and desensitization (13). **(A)** Desensitization of *in vitro* DNP-IgE sensitized mouse mast cells with DNP inhibits the release of granule mediators such as beta-hexosaminidase. Instead of one single optimal dose, 11 suboptimal sequential doses are given until reaching the optimal dose. **(B)** Desensitization of *in vitro* OVA-IgE sensitized mouse mast cells with OVA inhibits the release of granule mediators such as beta-hexosaminidase. Instead of one single optimal dose, 11 suboptimal sequential doses are given until reaching the optimal dose. **(C)** Desensitization of *in vitro* DNP-IgE sensitized mouse mast cell mediators with DNP inhibits the *de novo* generation of cytokines TNF alpha and IL-6 **(D)**. **(E)** Calcium entry (blue line) occurs after activation of DNP-IgE sensitized mouse mast cells with DNP but not when cells have been desensitized to DNP (red line, * DNP). Desensitization is specific since cells that were DNP desensitized and OVA-IgE sensitized presented calcium entry after OVA activation (red line, * OVA). **(F)** Membrane events are modified during desensitization with lack of internalization of desensitized antigens: left panels indicate that OVA desensitization does not present internalization of antigen (upper panel) as opposed to activation (lower panel with green labeled OVA internalized) and right panels indicate that after OVA desensitization another non desensitizing antigen such as DNP can be internalized (upper panel) as seen with activation (lower panel).

The mechanisms underlying drug desensitization are based on *in vitro* and *in vivo* models which have proposed that mast cells and basophils can be induced to predominantly inhibitory pathways by small incremental antigen doses, deactivating signal transduction and mediators release (13) (Figure 1).

Negative skin test is seen following desensitization in patients with IgE-mediated reactions, providing evidence of the powerful mechanisms which turn off skin mast cells (30–32). Partitioning of an optimal dose into 11–16 incremental doses starting at 1/1,000 the target dose and delivering them with sufficient time interval to mast cells; inhibits the acute release of beta-hexosaminidase, a mast cell granule mediator, prevents the generation of arachidonic acid and products such as leukotrienes and prostaglandins and the late generation of inflammatory cytokines (Figure 1) (13).

**Figure 2:**
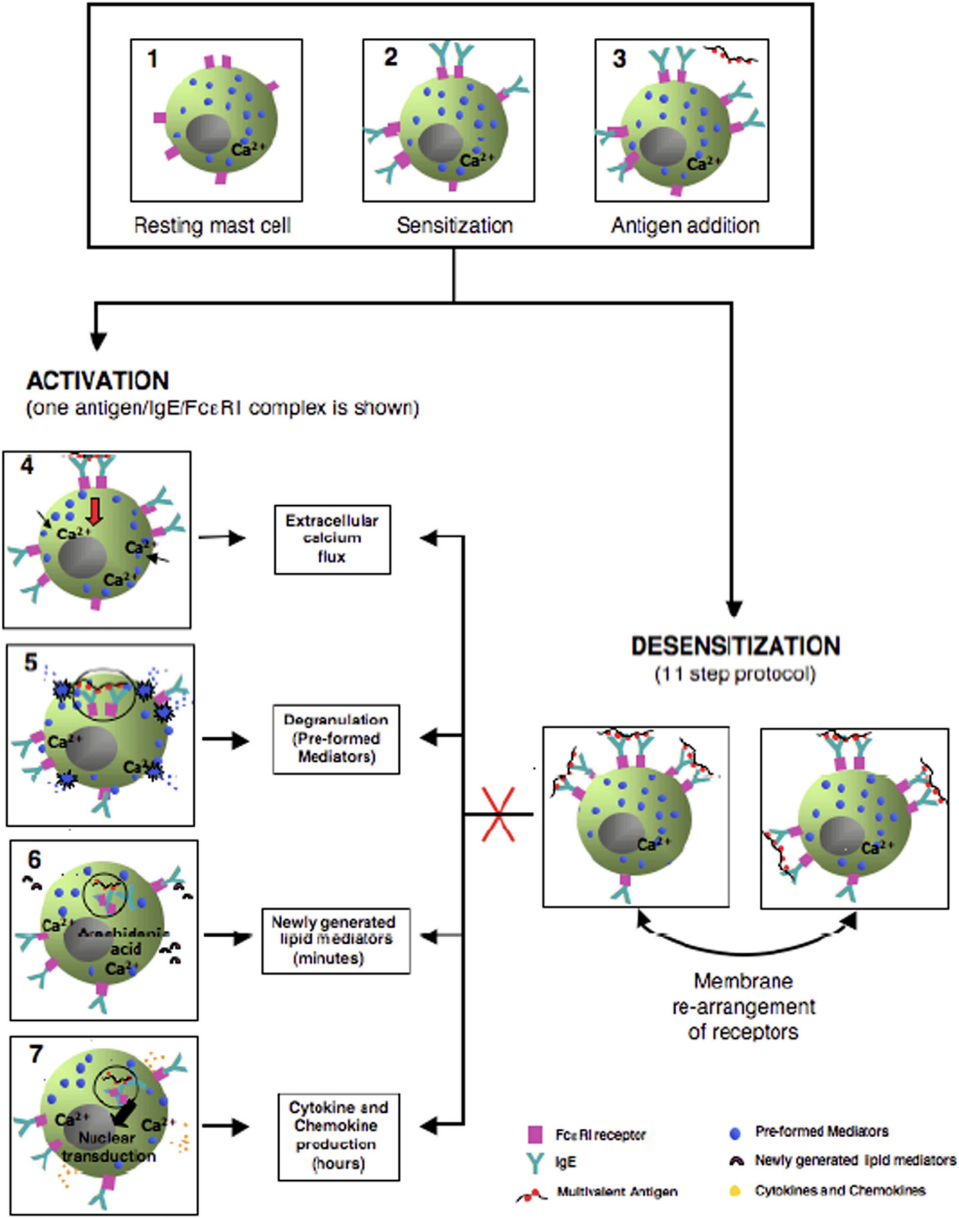
Model of *in vitro* mouse mast cells activation and desensitization. The left side cartoons provide the steps of antigen/IgE/FceRI activation starting from antigen cross-linking, internalization, calcium entry and release of granule mediators, generation of lipd mediators such as prostaglandins and leukotrienes, and production of late phase cytokines. The right sided panel provides the hypothetical membrane capping and rearrangement occurring during the delivery of sequential suboptimal doses of allergen in desensitization preventing internalization of antigen, calcium entry, and mediators release.

During desensitization, calcium influx is abolished and actin polymerization impaired, providing stability to intracellular granules in an antigen-specific fashion (Figure 1) (13). Membrane events that prevent internalization or modify its response to subthreshold doses of antigen occur during desensitization and are associated with incremental unresponsiveness to specific antigen (Figure 2) (13). Association of the FceRI to ITIM containing receptors, capable of dephosphorylating ITAMs receptors has been postulated as one of the mechanisms of desensitization. Mast cells desensitized to one antigen are responsive to a second non desensitizing antigen, providing evidence of compartimentalization and highly specialized and regulated intracellular processes (Figure 1) (13).

**Figure 3:**
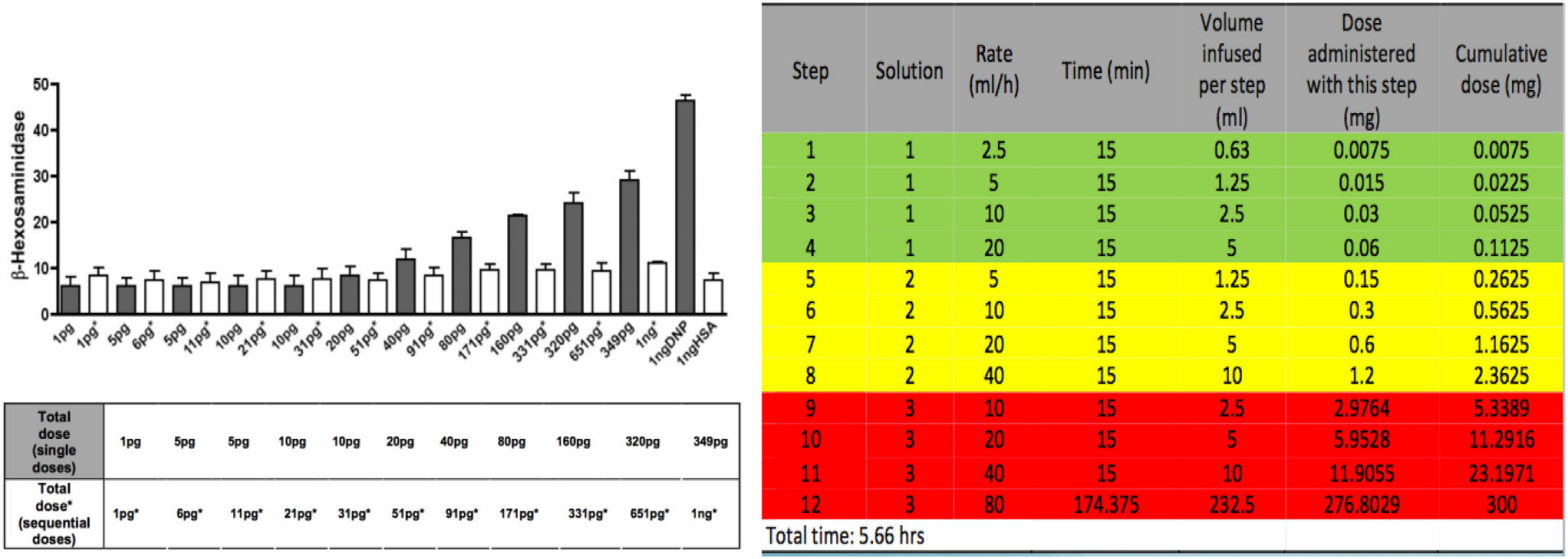
*In vitro* and *in vivo* protocols for induction of IgE/antigen desensitization. In the left panel in black, increasing single doses of antigen induce a dose response release of beta-hexosaminidase *in vitro* mouse mast cells. In white, the same doses given sequentially induce a profound inhibition of the beta -hexosaminidase release. In the right panel, a model of desensitization protocol used for human desensitization in which 3 bags and 12 steps (4 steps per bag) are used to administer sequential doubling doses every 15 min which provides the target dose of 300 mg after 5.66 h when the last step is completed.

**Figure 4:**
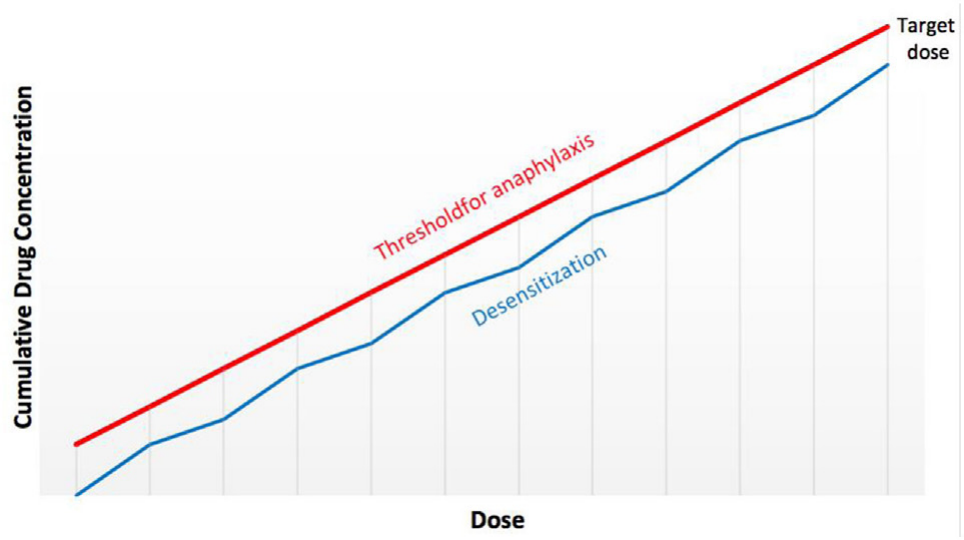
Putative mechanism of protection against anaphylaxis during human desensitizations. By delivering the target dose of the drug by small incremental doubling doses (Figure 3), the threshold for anaphylaxis is re-established at each step and never reaches that of the initial triggering dose.

The protocols used for *in vitro* desensitization have been adapted *in vivo* and further adaptations have produced safe protocols for human use (Figure 3) with similar dose increments and interval times (25–28). These human protocols have now been used in thousands of cases with remarkable safety since the inhibitory mechanisms of desensitization protect against anaphylaxis (Figure 4) (33, 34).

Whether after multiple desensitizations neutralizing antibodies can be generated which may block allergenic drug epitopes has been hypothesized (6). Maintaining drug desensitization state depends upon continued drug exposure and desensitized drugs require administration at regular intervals to maintain a stable pharmacokinetics state. Desensitization needs to be repeated if several half-lives of the medication have elapsed (6, 33).

**Figure 5:**
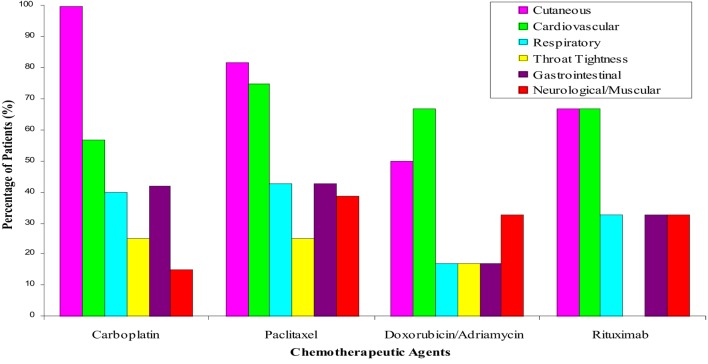
Symptoms and signs of hypersensitivity reactions amendable to desensitization. Carboplatin and other paltins such as cisplatin and oxaliplatin reactions include classical symptoms of anaphylaxis with cutaneous, respiratory, cardiovascular, and gastrointestinal symptoms. Reactions to taxenes including paclitaxel and docetaxel present with pain as a neuromuscular symptoms in up to 4% of the patients. Doxorubicin/adriamycin and other chemotherapies present with sudden onset hypo or hypertension in up to 60% of patients and rituximab and other monoclonal antibodies present with cutaneous and cardiovascular symptoms in 70% of the patients.

Protocols for drug desensitization have been successfully used for antibiotics, chemotherapy drugs, and MoAbs among other drugs in patients with IgE and non-IgE-mediated HSRs (Figure 5) (6, 14, 33, 35). The phenotypes of reactions amendable to desensitization include immediate and delayed reactions. Typical type I reactions usually begin within minutes of initiation of the infusion to few hours after the infusion due to anti-histamine and steroid premedication. The signs and symptoms include puritus, flushing, urticaria, angioedema, throat tightening, wheezing, nausea, diarrhea, hypotension, syncope, seizures, and cardiovascular collapse which can lead to death. Atypical symptoms include back, chest, or abdominal pain (such as seen with taxanes, oxaliplatin, MoAbs such as rituximab) (33, 35).

Delayed reactions are attributed to type IV reactions and can occur several days after the infusion and are typically limited to the skin with maculopapular rashes (36). Reactions that involve mucosal membranes and/or are associated with systemic symptoms are not amenable to desensitization due to the risk of inducing a severe systemic reaction with small amounts of drug antigen (16, 21, 29).

**Figure 6:**
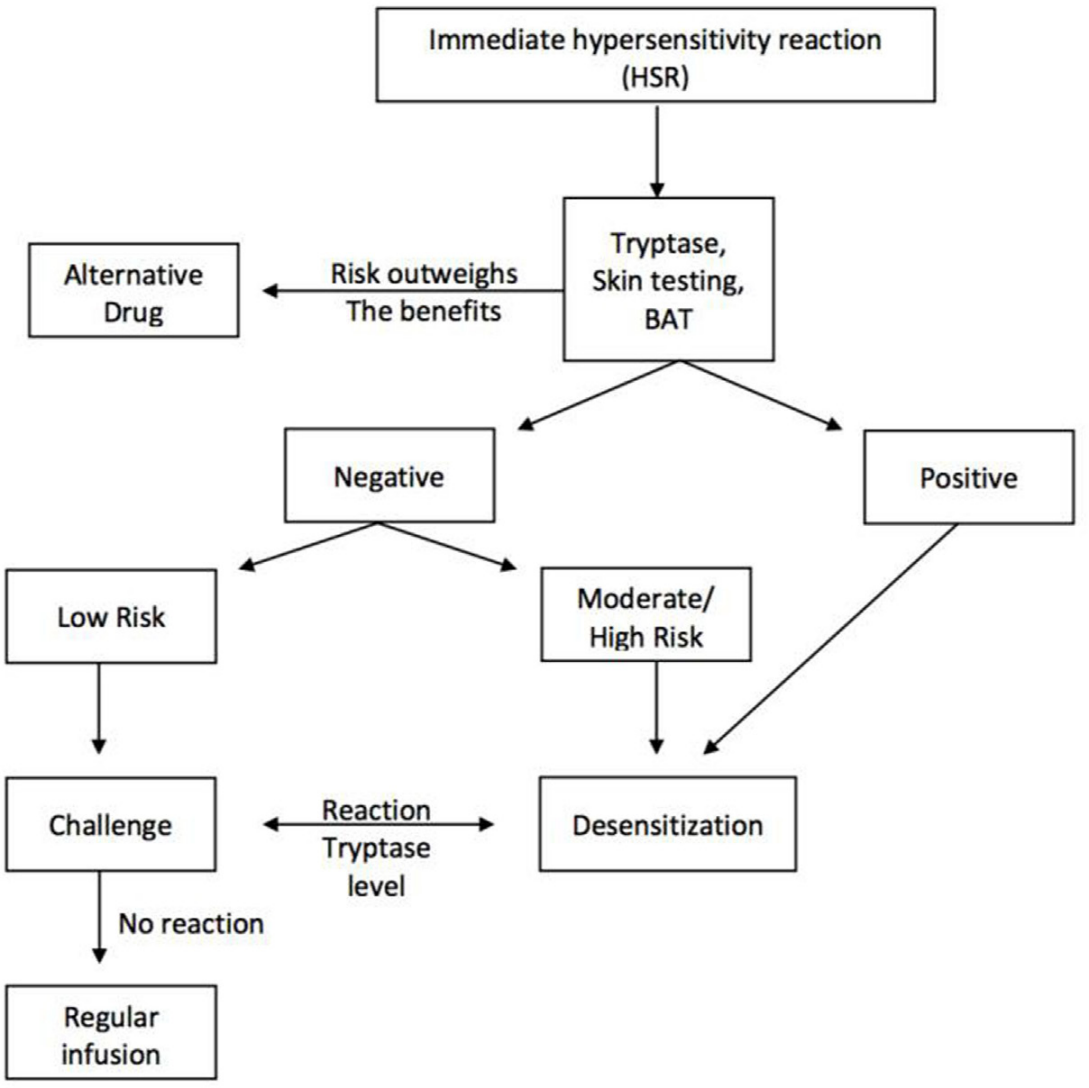
Algorithm for the evaluation of drug hypersensitivity reactions and the role of desensitization for the re-introduction of the first-line medications, when no alternative is available or the alternative does not provide the same benefits or life expectancy as the first line.

Desensitization should be considered in patients with reaction phenotypes consistent with type I and type IV reactions who have no alternative therapy or for whom alternative therapies are of less value or can induce more side effects. The algorithm for the evaluation of these patients is seen in Figure 6 (6, 34, 37). The nature and symptoms of the initial reactions needs to be established and tryptase and skin test provide evidence of IgE and/or mast cell involvement. BAT is a research tool and cannot be applied to current clinical practice.

Drug desensitization can be performed in patients of any age and in pregnant women when alternative therapies are not possible or when delaying therapy may incur a shortened life span. Anaphylaxis is the major risk during desensitization since patients are exposed to their allergic drug. Large series have demonstrated that most breakthrough reactions during desensitization are mild and less severe than the patient’s initial HSR and fatalities have not been reported, but all desensitizations can only be performed by an expert allergist familiar with the personalized protocol and potential reactions (Table 1) (17, 33, 35). The safety of desensitizations is paramount and patients with grade 3 severe initial reactions and anaphylaxis can be desensitized with minimal reactions as seen in Table 2.

**Table 2:** Safety of first desensitization in patients with grades 1, 2, and 3 initial reactions.

	First desensitization reaction grade				
Initial hypersensitivity reaction grade	0	1	2	3	Total
1	76 (61%)	38 (30%)	7 (6%)	4 (3%)	125
2	38 (58%)	22 (34%)	4 (6%)	1 (2%)	65
3	122 (60%)	54 (26%)	10 (5%)	19 (9%)	205
Total	236	114	21	24	396

*From Sloane et al. (33)*.

Desensitization is not recommended for type II and type III serum sickness-like reactions or in patients with reactions with skin desquamation, EM, Stevens–Johnson syndrome or toxic epidermal necrolysis, because small amounts of drug can induce irreversible and potentially fatal reactions (Table 1) (16, 21, 29).

**Figure 7:**
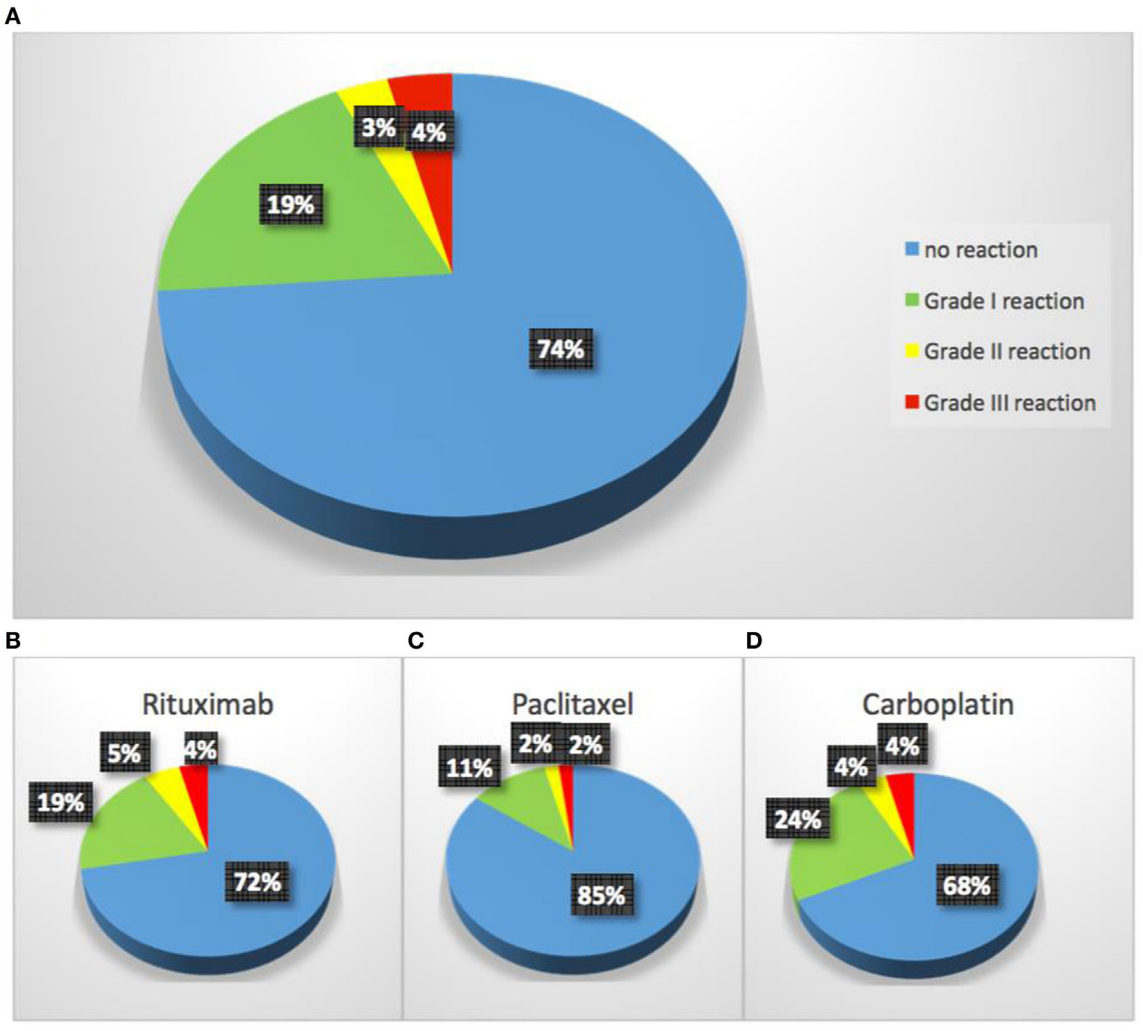
The overall safety of desensitization for common chemotherapy drugs and monoclonal antibodies.

The most commonly used intravenous desensitization protocols are standardized 12- to 16-step protocols modeled after *in vitro* protocols and can be personalized to all drugs with adjustment of the target dose, time intervals between doses and starting dose (Figure 3) (33, 38). Protocols are available for intravenous desensitizations but also for oral, subcutaneous, intraperitoneal, or intravenous routes in the outpatient and inpatient settings (39–41). Desensitization for delayed reactions is also available and may take several days but recent data suggest that some of these reactions may be amenable to shorter time intervals (8, 36, 42). The overall safety of desensitizations is similar for all medications provided the mechanism of the initial reaction is of type I, IgE and non-IgE or type IV. As seen in Figure 7, the overall safety indicates that 93% of patients present with no reaction or grade 1 reactions and all completed the desensitization.

Platins, taxanes, and MoAbs are the most common chemotherapy currently used in desensitization and are described below.

### Platin Hypersensitivity

Platinum compounds are used in ovarian, colorectal, endometrial, glioblastoma, lung, and pancreatic cancer as initial chemotherapy and in second-line or salvage settings. Carboplatin is the most popular since it is less nephrotoxic and neurotoxic than cisplatin. Allergic reactions to platins are IgE-mediated and require sensitization through multiple exposures, with 27% of women becoming allergic after seven life time exposures (12, 43, 44). Allergic symptoms typically start at the second round of treatment, when the cancer recurs and after 1–2 exposures sensitized patients present with flushing and pruritus which can progress to shortness of breath with further exposure and can lead to anaphylaxis, with hypotension and cardiovascular collapse (2, 4, 7, 33, 45). Patients bearing BRCA 1 and 2 gene mutations have an increased risk for carboplatin reactions, which can occur with fewer exposures (46, 47). Most reactions to platins occur during or shortly after the drug infusion and the phenotype is that of type I reaction. In a study of 60 carboplatin sensitized patients, 100% had cutaneous, 60% pulmonary, 40% respiratory, and 42% gastrointestinal symptoms (6).

The phenotype of reactions to oxaliplatin can be more complex with features including typical IgE-mediated symptoms and atypical symptoms such as back and pelvic pain and cytokine-mediated fever and chills (7, 11, 17, 48, 49). Antibody-mediated thrombocytopenia and immune complex-mediated syndromes with urticaria and proteinuria have also been observed (17, 50).

**Figure 8:**
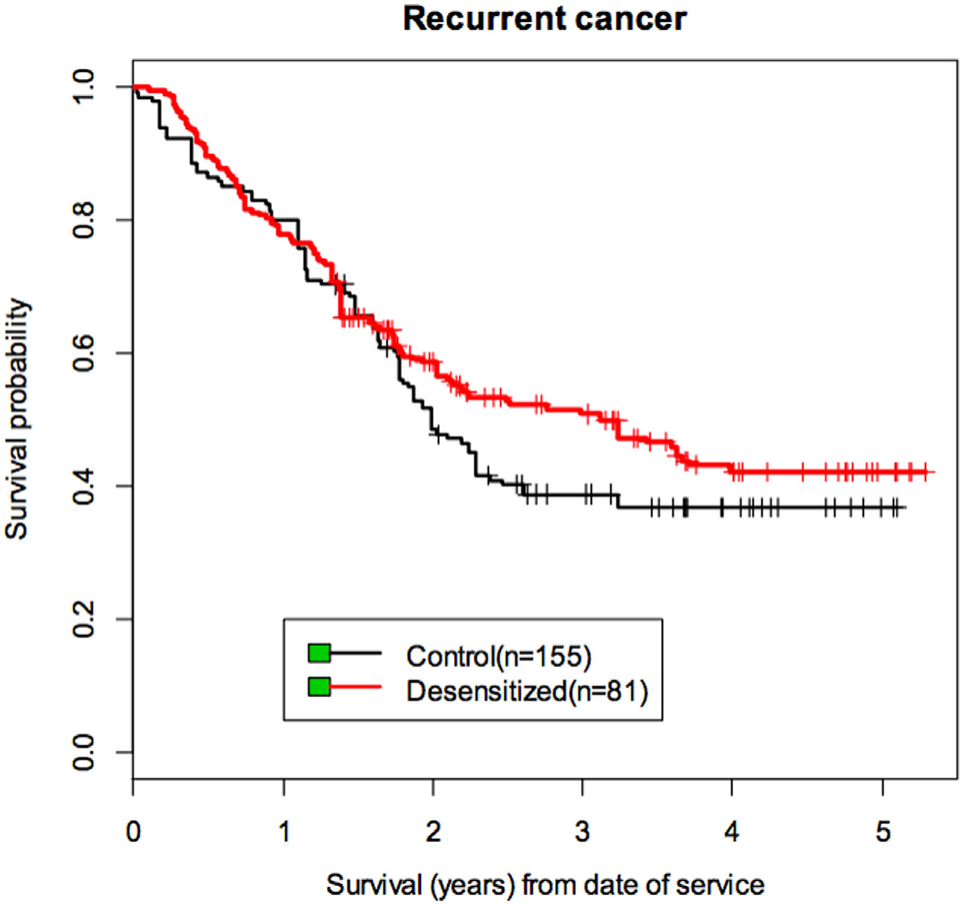
Life expectancy for cancer patients allergic and desensitized to carboplatin and non-allergic to carboplatin [from Sloane et al. (33)]. Allergic and non-allergic ovarian cancer patients treated with carboplatin or carboplatin desensitization presented a similar life expectancy with a non significant advantage for the allergy desensitized patients.

Skin testing to platins has been safely done (Table 3) and is diagnostic tool to demonstrate an IgE/mast cell mechanism in patients with carboplatin and cisplatin reactions (7, 11, 17). For patients exposed to six or more courses of carboplatin in the last 6 months the positive predictive value is up to 86% (11). Oxaliplatin skin testing is negative in up to 50% of patients presenting type I reactions, indicating other than IgE mechanisms or lack of skin test allergenic determinants (17). Circulating serum specific IgE has been demonstrated and patients reactive to oxaliplatin with detectable serum-specific IgE have also demonstrated IgE to carboplatin and cisplatin without exposure, indicating broad cross-reactivity (51). IgE to platins can be short lived since a study has demonstrated that ST is negative in a high proportion of patients with a remote history of HSR to carboplatin, but re-exposure leads to resensitization and severe reactions (11). When platins are considered as first-line therapy, desensitization is a safe option since increased premedications alone do not prevent anaphylaxis and cross-reactivity may prevent the use of other platins (51). Patients with severe cutanoues reactions, SJS and TEN are currently not candidates since the mechanism of the reactions is unknown and small amounts of medications may induce severe symptoms (1, 16, 21, 29). Desensitization provides a similar life expectancy as non-allergic non desensitized patients (Figure 8) without increased health costs (33).

**Table 3:** Skin testing for the diagnosis of chemotherapy drug allergy including platins, monoclonal antibodies, and paclitaxel.

Medication	Prick (mg/ml)	Intradermal (mg/ml)
Carboplatin	10	0.1, 1, 5, and 10
Cisplatin	1	0.1 and 1
Oxaliplatin	5	0.5 and 5
Rituximab	10	0.1, 1, and 10
Infliximab	10	0.1, 1, and 10
Tocilizumab	20	0.2, 2, and 20
Centuximab	20	0.2, 2, and 20
Traztuzmab	21	0.21, 2.1, and 21
Bevacizumab	25	0.25, 2.5, and 25
Cyclophosphamide	10	0.1, 1, and 10
Methotrexate	25	0.2, 2.5, and 25
Paclitaxel	1–6	0.001 and 0.01

### Taxanes

Taxanes are used in gynecologic, lung, breast, and prostate cancers and reactions to taxenes are among the most frequent chemotherapy reactions and fatalities have been reported (52). Paclitaxel and docetaxel have been the more frequently used and more recently other taxenes such as cabacitaxel and abraxene have become popular (53). Paclitaxel is an insoluble compound originally isolated from the bark of the pacific yew tree, Taxus Baccata tree and solubilized in cremophor and docetaxel is a semi-synthetic molecule derived from a precursor found in European yew tree needles and solubilized in polysorbate 80 (36, 54). The solvents can cause complement activation, generating anaphylotoxins C3a and C5a and leading to mast cell activation (55–57). Taxanes are used with premedications including anti-histamines and steroids due to a high rate of reactions in early clinical studies (54). The rate of reaction has decreased to less than 10% and typically occurs during the first or second lifetime exposure in up to 80% of the patients (54). The phenotype of the reactions include type I symptoms such as throat tightness, flushing, hypotension, and dyspnea but atypical symptoms such as chest, back, or pelvic pain (8, 36, 54).

Skin testing has uncovered IgE-mediated reactions to taxanes and a recent study reported that 103 of 145 taxane reactive patients (71%) had positive results. Negative skin test patients who were challenged were likely to tolerated taxane infusions without desensitization. Atopy was present in over 40% of the patients and because patients react at first or second exposure suggested prior sensitization or cross-reactivity with environmental allergens (8). Risk stratification based on biomarkers such as skin testing can safely guide the management to taxane reactions and allows a significant number of patients to resume regular infusions. For patients with positive skin test and significant initial reaction for whom taxanes are first-line therapy, desensitization should be considered (8).

### Monoclonal Antibodies

There are over 45 MoAbs currently in use for the treatment of cancer and inflammatory and autoimmune diseases. Reactions to MoAbs depend on their structure and vary from chimeric mouse-human, humanized, to fully human. Some of the most frequently used MoAbs are presented in Table 4, including their targets, incidence of overall injection/infusion site reactions, and HSRs (19, 34, 35).

**Table 4:** Common monoclonal antibodies in use and rate of overall reactions and hypersensitivity reactions (HSR).

Drug	Target	Overall reactions	HSR
Rituximab (Rituxan^®^) IV	CD20	77% (first infusion) (52)	5–10% (53)
Ofatumumab (Arzerra^®^) IV	CD20	44% (first infusion) (54)	2% (55)
		67% (combination therapy) (55)	
Obinutuzumab (Gazyva^®^) IV	CD20	66% (56,57)	(58)*
Trastuzumab (Herceptin^®^) IV	HER-2	40% (mild; first infusion) (59)	0.6–5% (60)
Cetuximab (Erbitux^®^) IV	EGFR	15–21% (61)	1.1–5% (62–65)
			14–27% (Southern USA) (43,66,67)
Tocilizumab (Actemra^®^) IV	IL-6 receptor	7–8% (68)	0.1–0.7% (68)
Infliximab (Remicade^®^) IV	TNF-α	5–18% (69)	1%* (69)
Etanercent (Enbrel^®^) SC	TNF-α	15–37% (70)	<2% (70)
Adalimumab (Humira^®^) SC	TNF-α	20% (71)	1% (71)
Golimumab (Simponi^®^) SC	TNF-α	4–20% (72,73)	Not reported
Certolizumab (Cimzia^®^) SC	TNF-α	0.8–4.5% (74,75)	Not reported
Brentuximab (Adcetris^®^) IV	CD30	12% (76)	(77–79)*
Bevacizumab (Avastin^®^) IV	VEGF-A	<3% (80)	Not reported
Omalizumab(Xolair^®^) SC	lgE	45% (81)	00.9–0.2% (81,82)

*<p= 0.05

Monoclonal antibodies immunogenicity depends on the human content but fully human MoAbs, such as adalimumab and ofatumumab can induce severe HSRs likely due to the glycosylation patterns *in vitro* and the generation of neo antigens (58). This is best exemplified in reactions to cituximab which can occur at first exposure in patients sensitized through tick bites to the mammalian oligosaccharide epitope, galactose-alpha-1,3-galactose (alpha-gal) (59).

The phenotypes of MoAbs reactions include limited infusion reactions, IgE-mediated reactions, serum sickness-like reactions, cytokine storm-like reactions, and mixed reactions. Infusion reactions are characterized by nausea, chills, fever, and malaise and for trastuzumab these reactions can occur in up to 40% of patients (34, 35). Like cytokine storm-like reactions, which are more severe, can associate with hypotension, oxygen desaturation, and require treatment with steroids and COX-1 inhibitors, proinflammatory cytokines (such as IL-6 and TNF-α) are thought to be involved (60, 61).

Immediate and delayed HSRs can occur with MoAbs and serum sickness-like reactions, such as seen with infliximab and omalizumab, which can present with rash, myalgia, fever, polyarthralgias, pruritus, edema, and fatigue (35).

Monoclonal antibodies used subcutaneously can elicit injection-site reactions few hours after the injection and persisting for several days. The phenotype of these reactions include local redness, warmth, burning, itching, urticaria, pain, and induration, varying in frequency from 0.8 to 4.5% with certolizumab to up to 45% with omalizumab (39).

Reactions to MoAbs can occur during the infusion and should prompt interruption of the treatment and the evaluation of tryptase and inflammatory cytokines to further understand the mechanism of the reactions. Skin testing with the offending agent can be done for type I and mixed reactions 2–4 weeks after the reaction to avoid false negative results, in particular in anaphylactic reactions in which natural desensitization can occur (35). An important consideration is cost of MoAbs; there are no available reagents at the present time for the evaluation of MoAb reactions and using a treatment vial may exceed several thousand dollars, precluding a diagnostic skin test evaluation. The negative predictive value of skin testing is not known and in a study of 23 patients reactive to trastuzumab, infliximab, or rituximab, 13 patients had positive skin test. Positive and negative skin test patient with significant initial reactions are candidates for desensitization and subcutaneous protocols are available. Successful desensitizations to rituximab, ofatumumab, obinutuzumab, trastuzumab, cetuximab, tocilizumab, infliximab, etanercept, adalimumab, golimumab, certolizumab, brentuximab, bevacizumab, and omalizumab have been reported (33–35).

## Conclusion

Drug hypersensitivity is an increasing health hazard, which can compromise the quality of life and the life expectancy of cancer patients and patients with chronic inflammatory diseases with reactions to their first-line therapy. Recognition of the mechanisms of the reactions into phenotypes, understanding the underlying endotypes and evaluation of biomarkers is key to personalized medicine and enhanced patient safety allowing for informed decisions regarding drug re-exposure and desensitization. Serum tryptase, skin testing, BAT, and specific IgE are helpful diagnostic tools which will be complemented in the future with genotyping to identify patients at risk before reactions occur. Appropriate treatment of the reactions including epinephrine use and management with personalized desensitization protocols can enhance the quality of life, life expectancy, and safety of an increasing at risk population of patients with cancer and inflammatory diseases allergic to their best medications. Most patients with reactions with phenotypes consistent with type I and type IV reactions are candidates for desensitization, which can provide advancement of personalized treatments. Drug desensitization protects against anaphylaxis and activates inhibitory mechanisms which need further research to uncover cellular and molecular players amendable to pharmacological applications, which can make desensitization safer and more effective.

## Author Contributions

All authors listed have made a substantial, direct, and intellectual contribution to the work and approved it for publication.

## Conflict of Interest Statement

The author declares that the research was conducted in the absence of any commercial or financial relationships that could be construed as a potential conflict of interest.
